# Pretreatment with S-Nitrosoglutathione Attenuates Septic Acute Kidney Injury in Rats by Inhibiting Inflammation, Oxidation, and Apoptosis

**DOI:** 10.1155/2021/6678165

**Published:** 2021-02-01

**Authors:** Heng Fan, Jian-wei Le, Min Sun, Jian-hua Zhu

**Affiliations:** Department of Intensive Care Unit, Ningbo First Hospital, Ningbo, Zhejiang Province, China

## Abstract

**Objective:**

We aimed to investigate the protective effect of s-nitrosoglutathione (SNG) pretreatment on acute kidney injury (AKI) in septic rats.

**Methods:**

We constructed a rat model of sepsis by cecal ligation and puncture and observed the survival of the rats. We obtained kidney and blood samples from rats, observed the pathological damage to the kidney tissues, and evaluated kidney function and the expression levels of inflammatory factors. We also detected the expression of induced nitric oxide synthase (iNOS) and cyclooxygenase-2 (COX-2) in the kidneys by immunohistochemistry and evaluated the apoptosis of kidney tubular epithelial cells (KTEC) by TUNEL.

**Results:**

Pretreatment with SNG significantly reduced the mortality of septic rats, attenuated kidney pathological damage, and decreased the levels of serum creatinine, plasma neutrophil gelatinase-associated lipocalin, and plasma kidney injury molecule-1. Moreover, SNG pretreatment decreased the levels of TNF-*α* and IL-1*β* in serum and kidney and reduced the expressions of NO, iNOS, PGE2, and COX-2 in the kidneys. Furthermore, pretreatment with SNG significantly reduced the apoptotic rate of KTEC and decreased the levels of caspase-3 and Bax mRNA, but increased the level of Bcl-2 mRNA.

**Conclusion:**

Pretreatment with SNG has a protective effect on AKI in septic rats, and the specific mechanisms are related to inhibition of inflammation, oxidation, and apoptosis.

## 1. Introduction

Acute kidney injury (AKI) is one of the common complications of patients with sepsis, and the incidence is increasing year by year [1]. Recent studies show that nearly 20 million people worldwide suffer from sepsis every year, and about 42% of the cases are complicated with AKI. The incidence increases at a rate of 1.5%-1.8% per year, with a mortality of 60% [2]. AKI is an independent prognostic risk factor for septic patients. Although medical technology has made significant progress in recent years, the overall mortality is not decreased [3]. Therefore, elucidating the pathogenesis of AKI caused by sepsis is an important aspect of research in this field.

In recent years, many studies have been carried out on the pathogenesis of septic AKI, and most of the studies focus on the mechanisms of anti-inflammation [4]. The inflammatory cascade that occurs after leukocyte activation leads to tissue microcirculation hypoperfusion, cellular ischemia, and hypoxia. Reduction of oxidative stress can effectively improve tissue microcirculation perfusion and oxygenation and avoid the development of multiple organ dysfunction [5]. Samuvel et al. [6] indicated that increase of nitric oxide (NO) is an early biomarker of AKI in septic patients, and NO synthesizes inducible nitric oxide synthase (iNOS), which leads to increased renal ischemia and hypoxia.

S-Nitrosoglutathione (SNG) is a synthesis material of NO, which can reduce the expression of iNOS and improve the microcirculation of the kidney [7]. Our previous study showed that SNG protects septic AKI by inhibiting inflammation, which is an effective drug for preventing the development of AKI [8]. However, the specific biological mechanism remains unclear. Therefore, in the present study, we used a rat model of sepsis to further explore the effect of pretreatment with SNG on the survival of rats and its regulation of oxidation and apoptosis.

## 2. Materials and Methods

### 2.1. Animals and Experimental Protocol

Male Sprague-Dawley (SD) rats (Animal Experimental Center of Zhejiang University, Hangzhou, China), 9 ± 1 weeks old, weighing 300 ± 50 g were used. They were kept under standard conditions (room temperature 22 ± 1°C, humidity 45-60%), given free access to food and drinking water, and fasted for 8 hours prior to the creation of the sepsis model. We used the method of cecal ligation and perforation (CLP) to construct the rat model of sepsis. Briefly, we anesthetized the rats with sodium pentobarbital (40 mg/kg; Zhejiang Chemical Factory, Hangzhou, China), made a 2-3 cm incision along the midline of the abdomen, pierced the cecum with a needle, squeezed the feces, and sutured the incision.

We randomly divided the rats into 4 groups of 10 rats each: sham operation group (control group), control+SNG (Yacoo Science Co. Ltd., Suzhou, China) group, CLP group, and CLP+SNG group. In the control group, we anesthetized the rats using sodium pentobarbital and made a 2-3 cm incision along the midline of the abdomen without piercing the cecum. In the CLP+SNG group, we administered SNG (50 mg/kg) to the rats intragastrically daily for one week, and the sepsis model was constructed after the last administration of SNG [8]. The rats were sacrificed by cervical dislocation 24 hours after the establishment of the model, and the blood samples and kidneys of the rats were obtained. All animal experimentation was conducted in accordance with laboratory animal operating standards and specifications and approved by the Experimental Animals Committee of the Ningbo University (No. AEWC-2017-33).

### 2.2. Survival Curves

We took 120 rats and divided them into 6 groups of 20 rats each: control group, control+SNG group, CLP group, CLP+SNG (12.5 mg/kg), CLP+SNG (25 mg/kg), and CLP+SNG (50 mg/kg) group. The timing was started after establishing the model of sepsis, the survival of rats was observed daily, and the end point was 7 days.

### 2.3. Histopathological Analysis

The rats were sacrificed by cervical dislocation 24 hours after establishing the model of sepsis. The kidneys obtained were fixed in 10% formalin, embedded and sectioned (2-3 *μ*m), and stained with hematoxylin-eosin (H&E). Two pathologists observed the histological sections under the light microscope (×400) by blind method. The pathological damages were scored according to the range and severity of kidney tubular epithelial cell damage: 0% = 0, 1–25% = 1, 26%–50% = 2, 51–75% = 3, and >75% = 4 [9].

### 2.4. Kidney Function Assessment

We assessed the kidney function by detecting the levels of serum creatinine (SCr; Böhde Co., Wuhan, China), plasma neutrophil gelatinase-associated lipocalin (pNGAL; Biyuntian Co., Shanghai, China), and plasma kidney injury molecule-1 (pKIM-1; Böhde Co., Wuhan, China) according to the manufacturers' instructions. The concentrations of SCr, pNGAL, and KIM-1 were calculated by a standard curve.

### 2.5. The Measurement of Inflammatory Cytokines, NO, and Prostaglandin E_2_ (PGE2)

According to the manufacturer's instructions, we used an enzyme-linked immunosorbent assay (ELISA) kit to measure the levels of TNF-*α* and IL-1*β* in serum and kidneys. In addition, we used the potassium nitrite concentration as a standard value to evaluate the nitrite production of the kidneys by the Griess Reagent System (Biotech Co., Ltd., Beijing, China) and used the method of ELISA to measure the concentration of PGE2 (Ningbo Chemical Factory, Ningbo, China).

### 2.6. Immunohistochemistry

We fixed the kidney in 10% formalin, sliced after paraffin embedding, dewaxed in xylene, dehydrated by an ethanol gradient, then placed in PBS buffer, and incubated at room temperature for 10 min. Primary antibody (iNOS, Invitrogen Co., Abnova, USA; cyclooxygenase-2 (COX-2), Solarbio Co., Beijing, China) and secondary antibody (iNOS, Invitrogen Co., Abnova, USA; COX-2, Solarbio Co., Beijing, China) were added, incubated overnight, DAB stained, dehydrated after hematoxylin counterstaining, xylene clarified, and solidified. Two professional pathologists observed the histological sections independently. Each slice was randomly selected from three fields under a 200-fold microscope magnification. We calculated the average integrated optical density (IOD) to determine the relative expressions of iNOS and COX-2 using the Video Pro 32 color image analysis system (Bio-Rad, California, USA).

### 2.7. TUNEL Staining

We used the method of TUNEL (Roche Co., Basel, Switzerland) to assess the apoptosis of kidney tubular epithelial cells (KTEC). The kidneys were embedded in paraffin, sliced (2 *μ*m), and dewaxed in xylene. Proteinase K was added, and the mixture was incubated for 12 min at room temperature. The sections were washed, TdT, FITC-dUTP, and FITC-dATP (Roche Co., Basel, Switzerland) added gradually, incubated for one hour in a humidified incubator, anti-FITC (Roche Co., Basel, Switzerland) added, and DAB stained. The number of apoptotic cells was observed under a 400-fold magnification under a light microscope, and three fields were randomly selected from each slice. We calculated the apoptotic rate of KTEC by dividing the positive cells by the total number of cells.

### 2.8. RT-qPCR

We extracted total RNA of the kidney by the TRIzol kit (Solarbio Co., Beijing, China) according to the manufacturer's instructions. We synthesized cDNA using RNA reverse transcription system (ILT Co., Darmstadt, Germany) and the following specific primers: capase-3 forward: 5′-CCA CGA TGC CGA TCA AAC TC-3′, reverse: 5′-ATC TGC AAC CGT TCC ATC AGC-3′; Bax forward: 5′-CGA ATT ACG GGC ATC TCG CCA-3′, reverse: 5′-CTC CCA CCT GCT CGC GTC-3′; and Bcl-2 forward: 5′-CGA CCT CTA GCC CGA TAA-3′, reverse: 5′-CTC CGG CTG AAC AGC ATC-3′. All the above primers were purchased from Jikai Company (Shanghai, China). We used a thermocycler to analyze the gene of interest (Eppendorf Co. Hamburg, Germany) and calculated the relative expression level of the gene of interest by the 2^-*ΔΔ*CT^ method.

### 2.9. Statistical Analysis

The statistical analyses were performed using GraphPad 6.02 software, and all values were expressed as mean ± SE or percentage. We assessed the differences between groups by one-way ANOVA and subsequent Tukey's post hoc tests and used the Kaplan-Meier method to analyze the survival of rats. *P* < 0.05 was considered statistically significant.

## 3. Results

### 3.1. Pretreatment of SNG on Mortality and Kidney Function of Septic Rats

**Figure 1:**
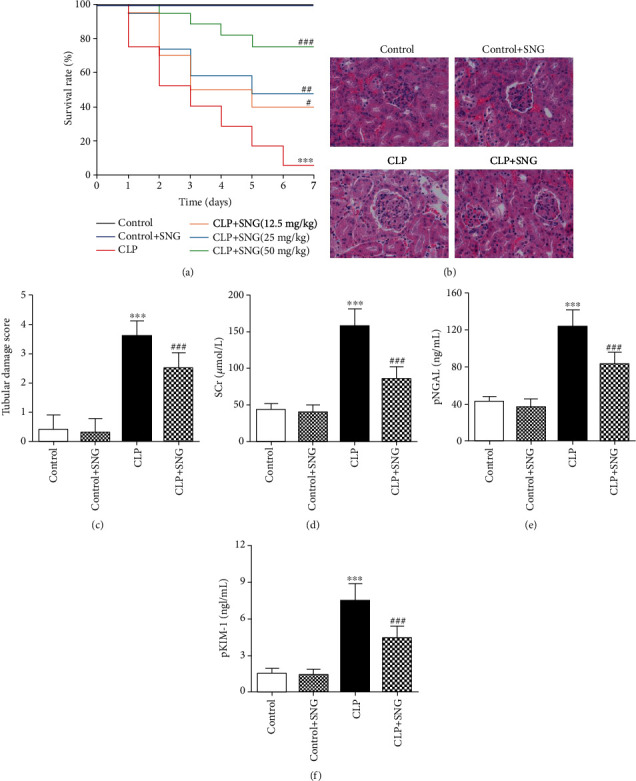
Effect of SNG pretreatment on mortality and kidney function. (a) The survival rate of rats in different groups (*n* = 20 in each group). (b) The pathological changes of kidney (H&E, ×400). (c) Estimation of the kidney tubular injury score. The levels of (d) SCr, (e) pNGAL, and (f) pKIM-1 in different groups. SNG: s-nitrosoglutathione; SCr: serum creatinine; pNGAL: plasma neutrophil gelatinase-associated lipocalin; pKIM-1: plasma renal injury molecule-1. Data are expressed as mean ± SD. ^∗∗∗^*P* < 0.001 vs. the control group; ^#^*P* < 0.05, ^##^*P* < 0.01, and ^###^*P* < 0.001 vs. the CLP group (*n* = 10 in each group).

To investigate the effect on mortality of septic rats, we plotted the survival curves. We found that the mortality of rats in the CLP group was significantly increased compared with the control group, and pretreatment with SNG significantly reduced the mortality of septic rats. Following comparison, we found that the 50 mg/kg dose of SNG pretreatment had the most significant protective effect on the 7-day survival of rats (Figure 1(a)). Therefore, we used SNG at a dose of 50 mg/kg for subsequent experiments.

Furthermore, in order to accurately assess the protective effect of SNG (50 mg/kg) pretreatment on the kidneys, we analyzed the pathological damage of the kidney by H&E staining. Our results showed that the structures of kidney tissues were normal in the control group and the control+SNG group, but in the CLP group, the structure was severely deformed; the KTEC were detached, the glomeruli were ruptured, and a large number of inflammatory cells infiltrated the kidney tissue parenchyma. However, in the CLP+SNG group, the structures of kidney tissues were intact, and a small amount of inflammatory cells infiltrated the interstitium (Figure 1(b)), but pretreatment with SNG significantly decreased the kidney tubular damage score compared with the CLP group (Figure 1(c)).

Then, we evaluated the kidney function of rats in each group. We found that the levels of SCr, pNGAL, and pKIM-1 were significantly increased in the CLP group, and SNG pretreatment significantly decreased the levels of SCr, pNGAL, and pKIM-1. Our results indicated that SNG pretreatment had a significant protective effect on kidney function of septic rats (Figures 1(d)–1(f)).

### 3.2. Effect of SNG Pretreatment on Inflammation in Septic Rats

**Figure 2:**
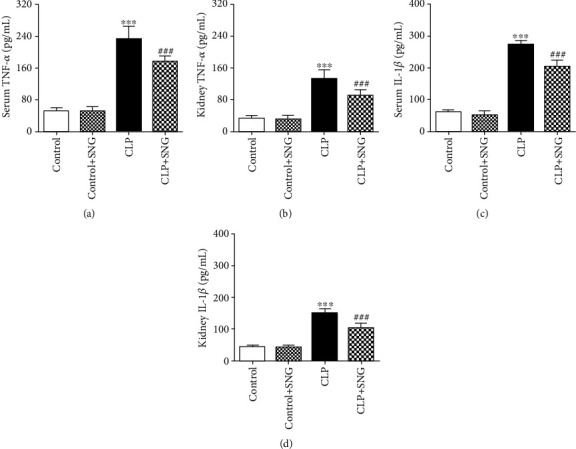
Effect of SNG pretreatment on inflammation. (a) The level of TNF-*α* in serum. (b) The level of TNF-*α* in the kidney. (c) The level of IL-1*β* in serum. (d) The level of IL-1*β* in the kidney. SNG: s-nitrosoglutathione; TNF-*α*: tumor necrosis factor-*α*; IL-1*β*: interleukin 1*β*. Data are expressed as mean ± SD. ^∗∗∗^*P* < 0.001 vs. the control group; ^###^*P* < 0.001 vs. the CLP group (*n* = 10 in each group).

Inflammation plays an important role in sepsis. Furthermore, we used the method of ELISA to detect the levels of inflammatory factors in serum and kidney. We found that the levels of inflammatory factors TNF-*α* and IL-1*β* were significantly increased in the serum and kidneys of the CLP group, and SNG pretreatment decreased the levels of TNF-*α* and IL-1*β* in serum and kidney (Figures 2(a)–2(d)). Our results indicated that SNG (50 mg/kg) pretreatment had a significant anti-inflammatory effect on septic rats.

### 3.3. Effect of Pretreatment with SNG on the Expression of iNOS and COX-2 in the Kidneys

**Figure 3:**
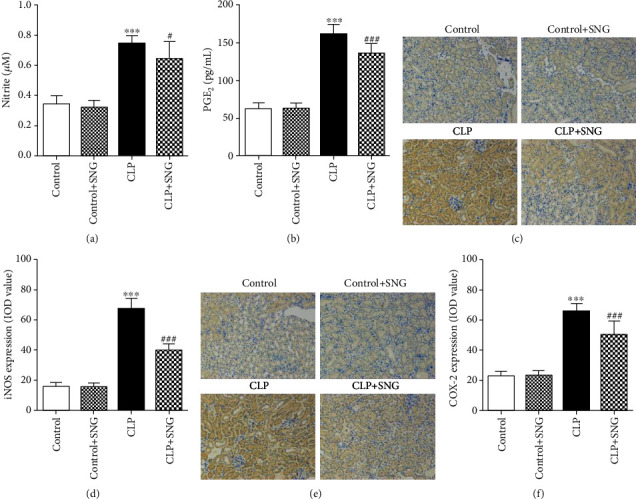
Effect of SNG pretreatment on the productions of NO and PGE2 and the expressions of iNOS and COX-2. The productions of (a) NO and (b) PGE2 in different groups; the effect of SNG on the expressions of (c, d) iNOS and (e, f) COX-2 (×200). SNG: s-nitrosoglutathione; NO: nitric oxide; PGE2: prostaglandin E_2_; iNOS: inducible nitric oxide synthases; COX-2: cyclooxygenase-2. Data are expressed as mean ± SD. ^∗∗∗^*P* < 0.001 vs. the control group; ^#^*P* < 0.05 and ^###^*P* < 0.001 vs. the CLP group (*n* = 10 in each group).

To determine the effect of SNG (50 mg/kg) pretreatment on oxidative stress in septic rats, we examined the expressions of NO and iNOS. We found that the expression of NO in the kidney was increased in the CLP group, and pretreatment with SNG significantly reduced the production of NO (Figure 3(a)). In addition, immunohistochemistry revealed that SNG pretreatment also significantly reduced the expression of iNOS in the kidneys (Figures 3(c) and 3(d)).

Since COX-2 acts as a downstream target of NO to promote PGE2 production, we further evaluated the expression levels of PGE2 and COX-2. We found that PGE2 production in the kidneys was increased in the CLP group, and pretreatment with SNG significantly reduced the production of PGE2 (Figure 3(b)). Then, we used immunohistochemistry to detect the expression of COX-2 in the kidneys. Consistent with the results of the above experiments, the expression of COX-2 was increased in the CLP group, and SNG pretreatment significantly decreased the expression of COX-2 (Figures 3(e) and 3(f)).

### 3.4. Effect of SNG Pretreatment on the Apoptosis of KTEC

**Figure 4:**
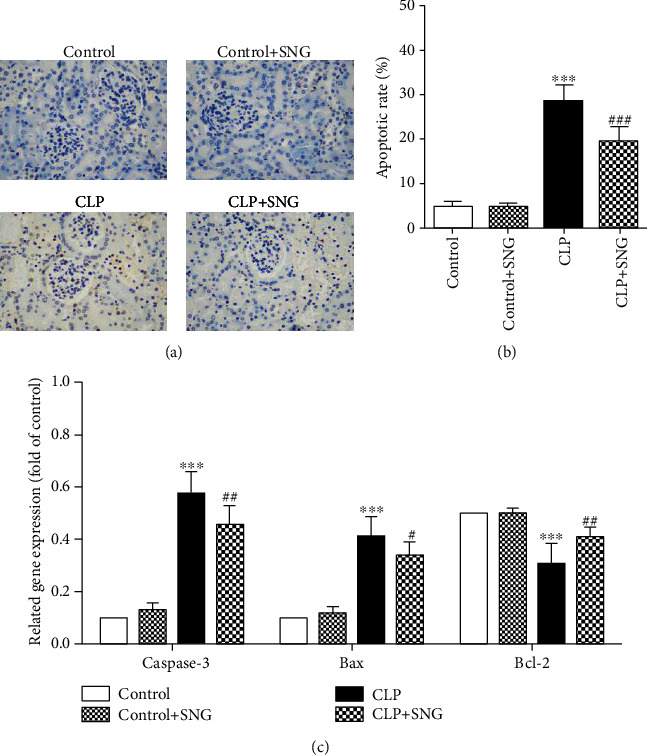
Effect of SNG pretreatment on the apoptosis of kidney tubular epithelial cells. (a) The apoptosis of kidney tubular epithelial cells was detected by TUNEL staining (×400). (b) The apoptotic rate was calculated from different groups. (c) Apoptosis-related proteins caspase-3, Bax, and Bcl-2 mRNA were detected by RT-qPCR. SNG: s-nitrosoglutathione. Data are expressed as mean ± SD. ^∗∗∗^*P* < 0.001 vs. the control group; ^#^*P* < 0.05, ^##^*P* < 0.01, and ^###^*P* < 0.001 vs. the CLP group (*n* = 10 in each group).

To investigate the effect of SNG (50 mg/kg) pretreatment on the apoptosis of KTEC, we used the TUNEL method to assess the number of apoptotic cells. As shown in Figures 4(a) and 4(b), positive apoptotic cells were stained dark brown, and the apoptotic rate was increased in the CLP group, while pretreatment with SNG significantly reduced the apoptotic rate. Moreover, we used RT-qPCR to detect the levels of caspase-3, Bax, and Bcl-2 mRNA in the kidneys. We found that the levels of proapoptotic factors caspase-3 and Bax mRNA were increased in the CLP group, and the apoptotic inhibitory factor Bcl-2 mRNA levels were decreased, while SNG pretreatment significantly decreased the levels of caspase-3 and Bax mRNA and increased the level of Bcl-2 mRNA (Figure 4(c)). Our results indicated that pretreatment with SNG had a significant inhibitory effect on the apoptosis of KTEC in septic rats.

## 4. Discussion

In mammals, NO is synthesized by endothelial cells, which has the function of regulating intercellular junction structure and cell signaling [10]. Moreover, NO is widely involved in a variety of biological activities, such as hypoxia, inflammation, and apoptosis [11]. SNG is a carrier of NO, which is fat soluble and can freely pass through vascular epithelial cells and endothelial cells, and participates in cellular inflammation and oxidative stress [12]. In the present study, to investigate the effects of SNG on the survival of septic rats and the specific protective mechanisms of AKI, we constructed a rat model of sepsis. Our results showed that SNG (50 mg/kg) pretreatment reduced the mortality of septic rats, and the specific mechanism might be related to anti-inflammatory, antioxidative, and antiapoptosis activity.

In recent years, the global medical technology has developed rapidly, but the mortality of patients with septic AKI remains high in the intensive care unit. Finding effective drugs and exploring specific molecular biology mechanisms are the key to solving these problems. In this study, we found that SNG pretreatment significantly attenuated the severity of kidney pathological damage and effectively improved kidney function, suggesting that pretreatment with SNG had a significant protective effect on kidney function.

It is well known that inflammation runs through the entire pathogenesis of sepsis, and the release of inflammatory factors can promote the occurrence and development of AKI [13]. In the early stage of sepsis, the proinflammatory mediators TNF-*α* and IL-1*β* are released and are highly toxic to the body [14]. Samuvel et al. [6] found that SNG has significant anti-inflammatory and antioxidant effects and a significant protective effect on septic AKI. In our previous study, we investigated the therapeutic effects of different doses of SNG on septic AKI by constructing a septic mouse model, and we found that 50 mg/kg of SNG pretreatment had the strongest protective effect on the kidneys [8]. Therefore, in the present study, we used 50 mg/kg SNG for the experiments, and we found that SNG pretreatment significantly reduced the levels of TNF-*α* and IL-1*β* in the serum and kidneys of septic rats, suggesting that SNG pretreatment effectively inhibited the inflammation.

In the early stage of sepsis, iNOS catalyzes the production of NO, and the release of large amounts of iNOS promotes the increase of immune cells, such as macrophages and neutrophils. Heemskerk et al. [15, 16] found that iNOS induces NO production and leads to oxidative stress in septic AKI, and iNOS inhibition inhibits NO production and effectively prevents kidney tubular epithelial cell damage. COX-2 is a downstream target of NO, which is positively correlated with the severity of the inflammatory response [17]. PGE2 is an enzyme product of COX-2, which also plays an important role in inflammation. In the present study, to investigate the effect of SNG pretreatment on oxidative stress in septic rats, we detected the levels of NO, iNOS, COX-2, and PGE2 in the kidneys. Our results showed that the levels of NO, iNOS, COX-2, and PGE2 were significantly increased in the kidneys of septic rats, while SNG pretreatment reversed these expressions, suggesting that SNG had an antioxidant effect on the kidneys of septic rats.

The role of apoptosis in the pathogenesis of septic AKI is a hot topic in current fields. Caspase-3 is a key signaling factor of the apoptosis pathway, which plays a critical role in the pathophysiology of apoptosis [18]. Bcl-2 inhibits cell apoptosis by weakening the permeability of mitochondrial membranes, generating free radicals, and inhibiting the release of cytochrome C [19]. Cunningham et al. [20] found that the apoptosis of KTEC is significantly increased in the early stage of sepsis, and inflammatory mediator-induced kidney cell apoptosis is an important mechanism of septic AKI.

To elucidate the effect of SNG on apoptosis of AKI in septic rats, we used the method of TUNEL to detect the apoptosis of KTEC. Consistent with the previous results, we found that the apoptotic rate of KTEC was significantly increased in the CLP group, while SNG pretreatment significantly reduced the apoptotic rate [6]. Furthermore, we used RT-qPCR to detect the levels of apoptotic signaling factors in the kidney. We found that the levels of proapoptotic factors caspase-3 and Bax mRNA were significantly increased, but the level of apoptotic inhibitor Bcl-2 mRNA was significantly decreased, and pretreatment of SNG reversed these phenomena. Our results confirmed that SNG pretreatment could effectively prevent apoptosis of KTEC and played a protective role on septic AKI.

## 5. Conclusions

By replicating a rat model of sepsis, we found that SNG pretreatment significantly reduced the mortality of septic rats, attenuated the severity of kidney tissue pathological damage, and improved kidney function. The specific mechanisms are related to inhibition of inflammation, antioxidation, and antiapoptosis.

## Data Availability

The underlying data supporting the results of our study can be found from the corresponding author.
